# Pelvic alignment changes during the perinatal period

**DOI:** 10.1371/journal.pone.0223776

**Published:** 2019-10-10

**Authors:** Saori Morino, Mika Ishihara, Fumiko Umezaki, Hiroko Hatanaka, Mamoru Yamashita, Tomoki Aoyama

**Affiliations:** 1 Department of Physical Therapy, Faculty of Comprehensive Rehabilitation, Osaka Prefecture University, Osaka, Japan; 2 Pilates Studio Wohl, Aichi, Japan; 3 Kishokai Medical Corporation, Aichi, Japan; 4 Department of Physical Therapy, Human Health Sciences, Graduate School of Medicine, Kyoto University, Kyoto, Japan; Mayo Clinic, UNITED STATES

## Abstract

**Background:**

The function of the pelvic bones is to transfer load generated by body weight. Proper function of the pelvic bones can be disturbed by alignment changes that occur during pregnancy. Further, misalignment of the pelvic bones can lead to pain, urinary incontinence, and other complications. An understanding of the timing and nature of pelvic alignment changes during pregnancy may aid in preventing and treating these complications.

**Objective:**

To investigate the changes in pelvic alignment during pregnancy and one month after childbirth.

**Methods:**

This is a prospective, longitudinal cohort study. Pelvic measurements were obtained for 201 women at 12, 24, 30, and 36 weeks of pregnancy, and 1 month after childbirth. The anterior and posterior width of the pelvis (the distance between the bilateral anterior superior iliac spines and the bilateral posterior superior iliac spines), the anterior pelvic tilt, and pelvic asymmetry (the mean left and right pelvic tilt degrees and the bilateral difference of the anterior pelvic tilt) were measured. For the change in pelvic alignment, a Friedman test was conducted to determine any significant difference in the measurements over time.

**Results:**

The anterior and posterior width of the pelvis became significantly wider with pregnancy progress and the anterior width of the pelvis at 1 month after childbirth remained wider than that at 12 weeks of pregnancy (p < 0.001). The anterior pelvic tilt increased during pregnancy and decreased after childbirth (p < 0.05).

**Conclusion:**

Some changes in pelvic alignment occur continuously during the perinatal period. Changes in the anterior width of the pelvis are not recovered at one month post-childbirth. Understanding these perinatal changes may help clinicians avert complications due to pelvic misalignment.

## Introduction

During pregnancy and after childbirth, significant changes occur to women’s bodies. Swelling of the abdomen with fetal growth and weight gain not only around the uterus but also in the whole body are major examples [1]. In addition, bone alignment changes, especially around the pelvis, occur in pregnancy [2,3]. A primary function of the pelvic bones is to transfer loads generated by body weight and gravity during activities of daily living [4]. This function is even more important during pregnancy because body weight increases over 10 kg in 40 weeks [5]. This important function requires that the pelvic bones are in a balanced position. Unfortunately, pelvic dislocation associated with pregnancy and childbirth has been reported [6].

The pelvis tilts forward as pregnancy progresses [1]. In addition, pregnancy-related hormones cause elasticity of the joints such as the sacroiliac joint, [7] and this increases the possibility of distortion of alignment. One previous study has reported a differing degree of pelvic anteversion in the right and left sides during pregnancy [8]. Multiple changes in pelvic alignment are closely related to pregnancy related ailments such as pelvic girdle pain [9]. For example, increased pelvic asymmetry during pregnancy is a risk factor for pregnancy-related sacroiliac joint pain [10]. The relatively small and flat sacroiliac joint of women compared with that of men, combined with the hormonal weakening of the ligaments and symphysis during pregnancy, may also lead to sacroiliac joint instability and pain [11]. Moreover, failed load transfer through the lumbopelvic region due to pelvic malalignment can cause low back pain [12,13] or loss of urethra closure and stress urinary incontinence [14]. Forward tilting of the pelvis is a risk factor for low back pain and pelvic girdle pain during pregnancy [15]. Furthermore, childbirth affects pelvic floor anatomy and increases the prevalence of urinary incontinence [16,17]. Accordingly, these changes significantly reduce the quality of life for many women.

Understanding the course of pelvic alignment changes during pregnancy and after childbirth is useful in providing medical advice for women. However, it is not clear when and how the changes of pelvic alignment occur. In our previous study, the differences in pelvic alignment among never-pregnant women, pregnant women, and postpartum women were investigated [18]. However, the study was not longitudinal in design and the changes in pelvic alignment during and after pregnancy were not assessed. A more thorough understanding of the timing and nature of gradual pelvic alignment changes and recovery during and after pregnancy may provide useful guidance for wellness during pregnancy and enable early detection of any pelvic abnormalities. Therefore, this study aimed to investigate the changes in pelvic alignment during pregnancy and after childbirth.

## Materials and methods

This study was part of a prospective, longitudinal cohort study that investigated the association between pelvic alignment and lumbopelvic pain during pregnancy. In the current study, the changes in pelvic alignment during pregnancy and after childbirth were investigated using the pelvic alignment information collected from participants in the longitudinal observational study. The present study was conducted in accordance with the guidelines of the Declaration of Helsinki, and the study protocol was reviewed and approved by the Ethics Committee of the Kyoto University Graduate School of Medicine (Approval number E2076). Written informed consent was obtained from all patients in accordance with the guidelines.

### Participants

Pregnant women were recruited at the obstetrics and gynecology clinics in Aichi Prefecture, Japan, between May 2014 and December 2014. The inclusion criteria were <12 weeks of pregnancy and a singleton pregnancy. Women with serious orthopedic disorders or neurological diseases such as after surgery for total hip arthroplasty or multiple sclerosis, respectively, were excluded. Those with high-risk pregnancies were also excluded. The obstetricians and midwives in the study’s implementation clinics checked whether the pregnant women fit the inclusion criteria or not for all pregnant women who came to the clinics for gynecological checkups. After being instructed about the study, particularly its purpose and methods, all participants gave written informed consent. In this way, 275 women who met the inclusion criteria for the survey and agreed to participate in the study were included. They were observed at 12, 24, 30 and 36 weeks of pregnancy and 1 month after childbirth. These periods were chosen for convenience to coincide with regular prenatal checkups.

### Questionnaire

Personal characteristics (age, height, and weight before the pregnancy), and number of previous deliveries were obtained at the time of recruitment. A copy of the questionnaire, both in English and in Japanese is given as S2 File. We also recorded weight at the time of survey, method of childbirth, and duration of labor.

### Pelvic alignment

**Fig 1:**
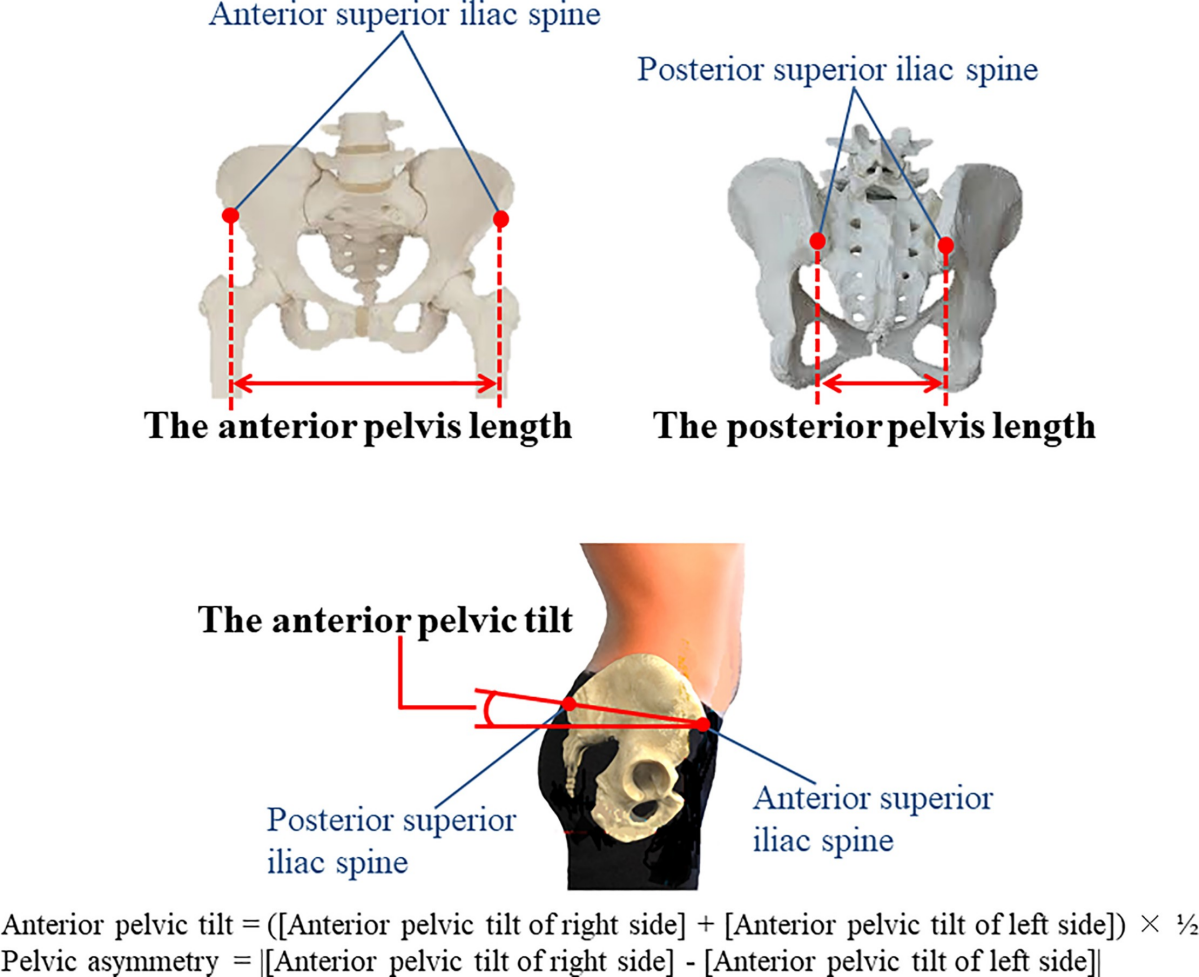
Measurement points for pelvic alignment.

Pelvic alignment was assessed at 12, 24, 30, and 36 weeks of pregnancy, and 1 month after childbirth. Pelvic alignment was measured using a palpation meter (Performance Attainment Associates, USA). The length of the anterior and posterior pelvis and anterior pelvic tilt were measured bilaterally by placing the caliper tips of the palpation meter in contact with the ipsilateral anterior and posterior superior iliac spines (ASIS and PSIS). This method is valid, reliable, and cost-effective for calculating any changes or asymmetry in the patient’s anatomy [19,20]. During the pelvic alignment measurements, the subjects removed their shoes and stood with hands crossed in front of their chests. The lengths between both ASIS and both PSIS were measured in centimeters. Left and right anterior pelvic sagittal tilting was measured in degrees. The lengths between both ASIS and both PSIS were defined as the anterior width of pelvis and posterior width of pelvis, respectively. The mean left and right pelvic tilt degrees and the bilateral difference in pelvic tilt were defined as anterior pelvic tilt and pelvic asymmetry, respectively (Fig 1). Fourteen midwives and physical therapists were trained as measurers and measured pelvic alignment of the women. Before taking the measurements, they learned the measurement method using the palpation meter and practiced repeatedly. In order to verify accuracy, nine primary measurers took separate measurements of pelvic alignment for one woman using the method outlined above. This verification procedure was repeated twice, two weeks apart. As a result, the measurement procedure showed acceptable intra- and inter-rater reliability with an Intraclass Correlation Coefficient (ICC) 1.1 of 0.989 (95% confidence interval (CI) 0.971–0.996) and an ICC 2.1 of 0.992 (95% CI 0.972–0.999) for the measurements of the anterior and posterior pelvis length, and an ICC 1.1 of 0.998 (95% CI 0.995–0.999) and an ICC 2.1 of 0.998 (95% CI 0.992–1.000) for the anterior pelvic tilt in this study.

### Statistical analysis

First, a normality test for all data was conducted to check if the data followed a normal distribution. Second, a Friedman test was conducted for each pelvic alignment measurement to determine any significant difference in the measurements over time. Third, Bonferroni correction for *p*-values was used to conduct the pairwise comparison of the 5 time points. Statistical analyses were performed using SPSS version 22.0 (SPSS, Chicago, IL, USA), with a significance threshold set at 0.05.

## Results

**Fig 2:**
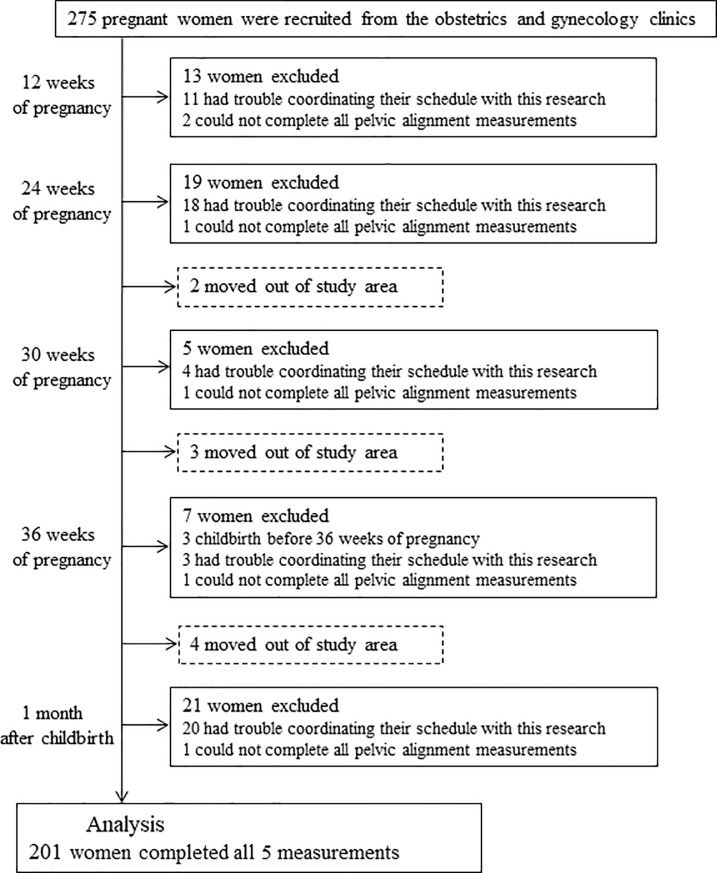
Flow diagram of the participants.

Participants with incomplete measurement data due to missed visits or delivery before 36 weeks of pregnancy were excluded from analyses. Complete data was obtained for 201 women. (Fig 2). Demographic characteristics of the participants are shown in Table 1. They were observed at 12 (12.9 ± 1.2 weeks), 24 (24.0 ± 0.9 weeks), 30 (30.0 ± 0.6 weeks), and 36 weeks of pregnancy (36.0 ± 0.3 weeks) and 1 month after childbirth (32.6 ± 5.6 days).

**Table 1:** Demographic differences of participants.

	Total
	(N* = 201)
Age (years)	30.9 ± 4.5
Height (cm)	158.5 ± 5.7
Body mass index before pregnancy (kg/m^2^)	21.0 ± 2.8
Weight before the pregnancy (kg)	52.8 ± 7.5
Weight at 12 weeks of pregnancy (kg)	53.2 ± 7.6
Weight at 24 weeks of pregnancy (kg)	57.4 ± 7.4
Weight at 30 weeks of pregnancy (kg)	60.1 ± 7.6
Weight at 36 weeks of pregnancy (kg)	62.5 ± 7.5
Weight at 1 month after childbirth (kg)	56.3 ± 7.6
Percentages of women with previous deliveries (%)	
Primipara	43.3 (N = 87)
Second child	38.8 (N = 78)
Third child	14.9 (N = 30)
Fourth child	2.0 (N = 4)
Fifth child	0.5 (N = 1)
Sixth child	0.5 (N = 1)
Percentage of people per method of childbirth	
Normal spontaneous vaginal delivery	81.1 (N = 163)
Forceps delivery	3.0 (N = 6)
Vacuum extraction delivery	2.5 (N = 5)
Caesarean section	11.9 (N = 24)
Epidural childbirth	1.5 (N = 3)

Age, Height, and Weight: Values are shown as mean ± standard deviation.

* N represents number of persons.

The data other than anterior width of the pelvis in 12, 30 and 36 weeks of pregnancy did not follow a normal distribution. Thus, the Friedman test as a non-parametric test with Bonferroni correction was conducted.

**Fig 3:**
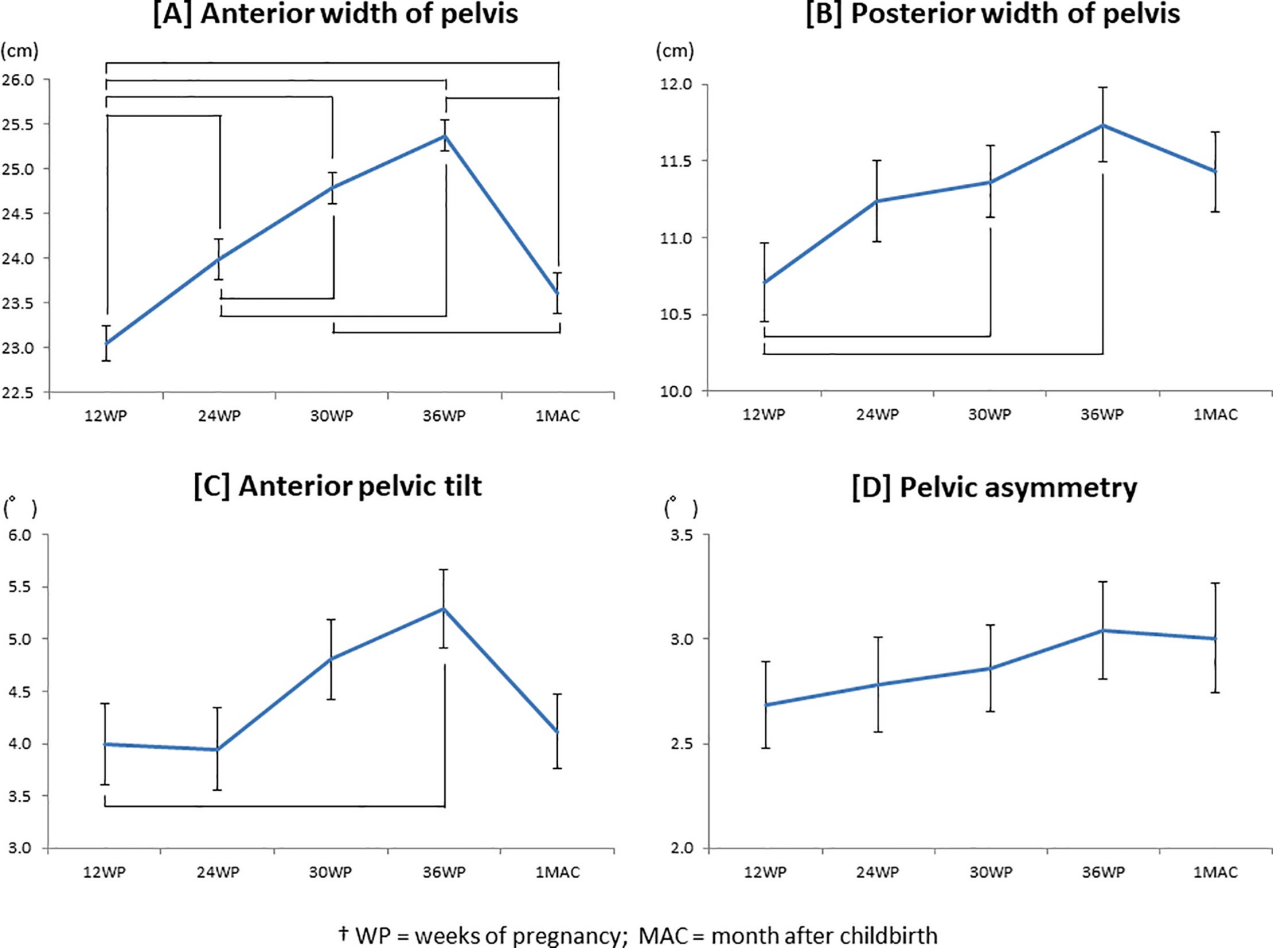
Change of pelvic alignment during and after pregnancy.

According to the Friedman test, pelvic alignment was significantly changed during pregnancy and after childbirth (Fig 3). The anterior width of the pelvis became significantly wider throughout the pregnancy period (12 vs. 24 weeks: 23.1 ± 2.8 cm vs. 24.0 ± 3.2 cm, respectively; *p* < 0.001, 24 vs. 30 weeks: 24.0 ± 3.2 cm vs. 24.8 ± 2.5 cm, respectively; *p* = 0.014, 30 vs. 36 weeks: 24.8 ± 2.5 cm vs. 25.4 ± 2.5 cm, respectively; *p* = 0.026) (Fig 3A). One month after childbirth, the anterior width of the pelvis was significantly narrower compared with week 24 (23.6 ± 3.1 cm vs. 24.0 ± 3.2 cm, respectively; *p* = 0.043), 30 (23.6 ± 3.1 cm vs. 24.8 ± 2.5 cm, respectively; *p* < 0.001) and 36 of pregnancy (23.6 ± 3.1 cm vs. 25.4 ± 2.5 cm, respectively; *p* < 0.001). On the other hand, the anterior pelvic width at one month post-delivery was significantly wider than that at 12 weeks of pregnancy (23.6 ± 3.1 cm vs. 23.1 ± 2.8 cm, respectively; *p* = 0.009).

There were no significant differences in the posterior width of the pelvis between weeks 12 and 24 (10.7 ± 3.6 cm vs. 11.2 ± 3.7 cm, respectively; *p* = 0.403), 24 and 30 (11.2 ± 3.7 cm vs. 11.4 ± 3.3 cm, respectively; *p* = 1.00), and 30 and 36 (11.4 ± 3.3 cm vs. 11.7 ± 3.5 cm, respectively; *p* = 1.00) (Fig 3B). The increase in posterior width was significant between weeks 12 and 30 (10.7 ± 3.6 cm vs. 11.4 ± 3.3 cm, respectively; *p* = 0.034) and between 12 and 36 (10.7 ± 3.6 cm vs. 11.7 ± 3.5 cm, respectively; *p* < 0.001). At 1 month after childbirth, there was no significant difference in the posterior width of the pelvis compared with that during pregnancy.

The anterior pelvic tilt increased during pregnancy and had decreased by one month after childbirth. There were significant differences between 12 and 36 weeks of pregnancy (3.99 ± 5.53° vs. 5.29 ± 5.33°, respectively; *p* = 0.037) (Fig 3C). Some pelvic asymmetry was observed throughout the investigational period (Fig 3D). The asymmetry increased slightly during pregnancy, but the difference during pregnancy and after childbirth was not significant.

## Discussion

The current study investigated the changes in pelvic alignment during pregnancy and one month after childbirth. The results document continuous changes in pelvic alignment during this period.

Specifically, both the anterior and posterior pelvic joints continually open during pregnancy. The pelvis is known to open due to joint relaxation and swelling of the abdomen with fetal growth during pregnancy [1,21]. Although this opening is necessary for fetal growth and delivery, pelvic recovery after childbirth is required to avoid future problems such as pelvic organ prolapse [22]. According to our results, recovery is not complete even at 1 month after childbirth. The anterior width of the pelvis may be slower to recover than the posterior width. Generally, it is desirable that the anterior and posterior recover simultaneously in order to avoid body dysfunction. For example, instability of the pubic symphysis is a severe symptom that may require surgery [23]. The widening of the pelvis after childbirth might be related to pelvic organ prolapse [24]. Thus, various pelvic belts are used to augment pelvic stability via external compression and additional closure forces in lumbopelvic disorders where stability is compromised [25–27]. Moreover, the widening of the anterior pelvis itself is sometimes the cause of pelvic dysfunction such as a gapping joint [28]. To make matters worse, these pelvic dysfunctions caused by pregnancy continue long term as chronic pelvic or low back pain [29]. Therefore, we especially need to promote recovery in the anterior width of the pelvis after childbirth in order to reduce and prevent long-lasting disorders that lower the quality of life beyond the perinatal period.

Our results support the general consensus that the pelvis tilts forward with pregnancy progress and does not fully recover after childbirth. Forward pelvic tilting is a risk factor for low back and pelvic pain during pregnancy [15]. According to our results, clinicians and researchers need to pay attention to pain caused by pelvic tilting, especially in late pregnancy because the pelvic tilt was significantly larger than that seen at 12 weeks of pregnancy. Recently, pelvic asymmetry is gaining attention for its relationship to some physical disorders such as pelvic pain [30, 31]. According to the results of the current study, pelvic asymmetry does not change significantly during and after pregnancy. However, asymmetry of pelvic alignment does exist, and cannot be ignored during the perinatal period when elasticity of the joints increases [7]. Further investigation with more participants is needed to assess the pelvic changes in women who experience pelvic asymmetry during and after pregnancy.

There were several limitations to the current study. First, the measurement of pelvic alignment of the participant was sometimes conducted by same measurer. Thus, the measurer might have remembered and been influenced by the previous result. Second, the timing of the measurements was not at equal intervals because visits were scheduled to coincide with the standard Japanese health checkups for pregnant women. Pre-pregnancy evaluations could not be standardized, and longer term follow-up after childbirth is necessary to fully assess pelvic recovery time. In addition, other factors that may affect pelvic alignment, such as the level of pregnancy-related hormones, daily activity, and job, were not investigated. However, despite these limitations, the changes in pelvic alignment during pregnancy and one month after childbirth were documented in this study. The cut-off value for recognizing pelvic alignment as abnormal has not been established. Thus, the current study was conducted to determine a reference value for abnormal pelvic alignment. Unfortunately, the data may not be sufficient for deciding the cut-off values. However, if the change of the pelvic alignment was markedly different from the results of the present study, it might be abnormal. Furthermore, future studies with a larger number of participants that limit the participants to being either primigravid or multigravid will provide more useful information. This is one of the few studies that have shown perinatal changes in pelvic alignment, and the results may help healthcare providers to prevent and treat complications due to pelvic opening, misalignment, and delayed postnatal recovery.

## Conclusions

In the current longitudinal study, the changes in pelvic alignment during and after pregnancy were investigated. The results demonstrated that both the anterior and posterior width of the pelvis become significantly wider with pregnancy progress. The anterior width of the pelvis is not recovered at 1 month after childbirth, and it is still wider than that at 12 weeks of pregnancy. The anterior pelvic tilt increases during pregnancy, and especially from 12 weeks to 36 weeks of pregnancy, and then decreases 1 month after childbirth. Pelvic asymmetry was found to be present throughout during pregnancy and after childbirth, though no significant change observed. From the results of the current study, the average values and changes of pelvic alignment throughout the perinatal period were revealed. The data of the women that could have had special effects on pelvic alignment were excluded in the present study. Although it is difficult to declare the cut-off values, the data of the current study might be useful as a reference to check the change of the pelvic alignment in perinatal periods. If the trend of change of pelvic alignment of a pregnant woman was clearly different from the data of this study, the approach for reforming the alignment is still useful for avoiding perinatal related dysfunction. Consequently these details on pelvic alignment during and after pregnancy might be useful for assessing the perinatal risk of lumbopelvic disorders in women and for developing appropriate treatments for pelvic misalignment based on the time when any alignment changes occur.

## Supporting information

S1 File STROBE Statement. Checklist of items that should be included in reports of cohort studies.
(DOC)

S2 File Questionnaire. A copy of the questionnaire used, both in English and in Japanese.
(DOC)
