# Supplementary material for: Pelvic alignment changes during the perinatal period
Source: PLoS One. 2019 Oct 10;14(10):e0223776. doi: 10.1371/journal.pone.0223776 (PMC6799872; doi:10.1371/journal.pone.0223776)
Supplement: S2 File — A copy of the questionnaire used, both in English and in Japanese. (DOC) [file pone.0223776.s002.doc]

マタニティチェックシート（初回）

記入日 月 日 氏名 妊娠第 週

**A. 基本的な背景についておたずねします。**

年齢： 歳 身長： cm 体重：妊娠前 kg/現在 kg

これまでの出産経験回数： 回

Maternity check sheet（first time）

Date (Month)/ (Day) Name Week number in pregnancy:

**A. Please answer some questions about your basic information**

Age： years Height： cm Weight：before the pregnancy kg/now kg

Number of previous deliveries：
